# Incidence and Mortality of Uveal Melanoma in Hungary: A Nationwide Study

**DOI:** 10.3390/cancers16050931

**Published:** 2024-02-25

**Authors:** Gábor Tóth, Béla Muzsik, Attila Szajkó, Pál Kerber, Elek Dinya, Béla Csákány, Zoltán Zsolt Nagy, János Németh

**Affiliations:** 1 Department of Ophthalmology, Semmelweis University, Mária utca 39, H-1085 Budapest, Hungary; csakanybela@gmail.com (B.C.); zoltan.nagy100@gmail.com (Z.Z.N.); nemeth.janos@med.semmelweis-univ.hu (J.N.); 2 National Directorate General for Hospitals, Diós árok 3, H-1125 Budapest, Hungary; muzsik.bela@okfo.gov.hu (B.M.); szajko.attila@okfo.gov.hu (A.S.); kerber.pal@okfo.gov.hu (P.K.); 3 Institute of Digital Health Sciences, Semmelweis University, Ferenc tér 15, H-1094 Budapest, Hungary; dinya.elek@public.semmelweis-univ.hu

**Keywords:** uveal melanoma, incidence, mortality, Hungary, Central and Eastern Europe

## Abstract

**Simple Summary:**

Although rare, uveal melanoma is the most common primary malignant ocular tumour in adults. Despite developments in the local treatment of uveal melanoma (such as proton beam therapy and brachytherapy) and the systemic treatment of metastatic uveal melanoma (such as chemoimmunotherapy and protein kinase inhibitors), no extended survival has been reported during the last few decades. The incidence of uveal melanoma differs between countries because of differences in ethnicities. White people may be more likely to develop uveal melanoma. Several studies have reported the epidemiology of uveal melanoma in Western Europe and worldwide. However, no studies have published data on uveal melanoma, neither in Hungary nor in Central and Eastern Europe. This study aimed to estimate the incidence and all-cause mortality of uveal melanoma in the adult population in Hungary.

**Abstract:**

Uveal melanoma (UM) is the most common primary malignant ocular tumour in adults, although its epidemiology in Central and Eastern Europe is unclear. This study aimed to analyse the incidence and all-cause mortality of UM in Hungary. This nationwide, retrospective, longitudinal study used data from the National Health Insurance Fund and included patients aged ≥18 years who were newly diagnosed with UM (ICD-10 C69.3 or C69.4) between 1 January 2012 and 31 December 2021. Age-standardised incidence and all-cause mortality rates were calculated using European Standard Population data from 2013. We identified 88 and 70 new patients with UM in 2012 and 2021, respectively, showing an almost stable trend. Age-standardised incidence rates varied between 6.40 and 10.96/1,000,000 person-years (PYs) during the analysed period. The highest age-standardised incidence was detected among men (13.38/1,000,000 PYs) in 2015. All-cause mortality decreased from 4.72/1,000,000 PYs to 0.79/1,000,000 PYs between 2012 and 2021. In conclusion, the UM incidence rate in Hungary is comparable to European incidence rates. The incidence did not markedly change, whereas all-cause mortality decreased during the study period, but this decline could not be attributed to improved treatment modalities for primary tumours and metastatic UM.

## 1. Introduction

Uveal melanoma (UM) is the most frequent primary malignant ocular tumour [1,2] and carries a substantial risk of visual impairment and mortality due to metastatic disease [3]. UM is a rare cancer, representing 3.2% of all melanoma cases [4]. Its incidence is approximately 6.6 per million people in Europe [5]. Shields et al. [6] reported that 4% of UM occurs in the iris, 6% in the ciliary body, and 90% in the choroid. Small-sized and peripherally located tumours may be asymptomatic and can easily be overlooked during routine eye examinations, leading to delayed diagnosis, loss of the eye, and potential metastasis [7]. The most frequent site of metastasis is the liver, which may develop even 20 years after initial diagnosis [8,9].

Despite developments in the treatment of UM, such as brachytherapy and proton beam therapy, and metastatic UM, including chemoimmunotherapy, protein kinase inhibitors, hepatic intra-arterial chemotherapy, isolated hepatic perfusion, and immunoembolisation, no extended survival data have been reported in the last few decades [4]. Enucleation remains the preferred procedure for treating large UMs [3].

Several studies have reported the epidemiology of UM in Western Europe and other regions [2,5,10,11,12,13,14,15,16]. However, no studies have published data on UM in Hungary or Central and Eastern Europe. Therefore, epidemiological surveys are needed to estimate the incidence and mortality of UM in this region.

National population-based databases offer the most accurate data on cancer incidence and mortality [11]. The National Health Insurance Fund (NHIF) database-based epidemiological research technique is a reliable and valid survey method for Hungary, as demonstrated in recent years [17,18,19]. Our study aimed to estimate the incidence and all-cause mortality of UM in people aged 18 years and older in Hungary.

## 2. Materials and Methods

### 2.1. Study Design

We used the database of the NHIF, a primary public healthcare financing company in Hungary. The NHIF database represents almost 100% of the Hungarian population and includes data on patient demographics, medical diagnoses coded according to the International Classification of Diseases 10 (ICD-10) system, and medical procedures coded according to the International Classification of Health Interventions (ICHI). The NHIF finances 100% of UM-related ophthalmic interventions as no other insurance system covers UM treatment in Hungary.

This study was approved by the Regional and Institutional Committee of Science and Research Ethics of Semmelweis University (Budapest, Hungary) (no. 272/2023). This study was conducted in accordance with the Declaration of Helsinki of 1975, revised in 2013.

This nationwide, retrospective, longitudinal study included all patients aged 18 years and older at the time of diagnosis who were newly diagnosed and treated for UM—ICD-10 code: C69.3 (choroidal melanoma) or C69.4 (ciliary body melanoma) between 1 January 2012 and 31 December 2021 in Hungary. Treatment with ruthenium-106 plaque brachytherapy (ICHI code: 01502) or enucleation (ICHI code: 51630) as a medical intervention was required for inclusion to identify new cases, as other medical interventions are not available as monotherapies for UM in Hungary. To identify patients with newly diagnosed UM in 2012, a period of two years between 2010 and 2011 was considered as the reference period.

Data were anonymised during collection, and only non-identifiable data were processed for analysis.

The incidences of newly diagnosed UM cases and all-cause mortality rates were represented as crude numbers and age-standardised rates. The NHIF database does not contain cause-specific mortality data; thus, all-cause mortality was analysed in the UM population. The prevalence was calculated based on the annual number of people with UM who were alive on 1 January of the given year. Patients newly diagnosed with UM in a given year were also included in the annual prevalence analysis.

All-cause mortality was calculated based on the number of people who died among patients with UM between 1 January and 31 December of a given year.

Population size data for Hungary given by age and gender for standardisation were obtained from the public annual reports of the Hungarian Central Statistical Office (HCSO) [20]. Age-standardised rates [per 1,000,000 person-years (PYs)] were calculated from crude incidence and all-cause mortality numbers using the European Standard Population 2013 to facilitate comparisons with earlier studies.

We analysed the total and annual changes in the incidence and all-cause mortality of UM in Hungary.

### 2.2. Statistical Analysis

The population sizes of Hungary were calculated based on the mid-term population sizes for each year published by the HCSO. Changes in age-standardised incidence and all-cause mortality rates of UM over time were analysed using Poisson regression. Annual mean changes in the incidence and all-cause mortality rates were determined using a regression model with a 95% confidence interval (95% CI). Between 2012 and 2021, the number of occurrences of UM was considered the outcome, and the logarithm of the mid-term population size was used as the offset variable in the regression model. The year served as the explanatory variable. We also calculated the incidence and all-cause mortality rates with the corresponding 95% CI values for each year, as well as gender comparisons between the incidence and all-cause mortality rates. The CI values were calculated according to the recommended method provided by Altman et al. [21]. The incidence and population data of the NHIF in Hungary were standardised using HSCO data for the examined period (2012–2021). Standardisation calculations were based on the typical methodological foundations published by Jensen et al. and dos Santos Silva [22,23]. Using the Pearson chi-square test, we examined the changes in mortality between the first (2012) and last (2021) years using a 2 × 2 contingency table (row = year; column = dead or live). Statistical significance was set at *p* < 0.05. All calculations were performed using SPSS software (version 28.0.1.0, IBM, Armonk, NY, USA) and Medcalc online calculator version 22.016 (MedCalc Software, Ostend, Belgium).

## 3. Results

### 3.1. Crude Numbers

**Figure 1:**
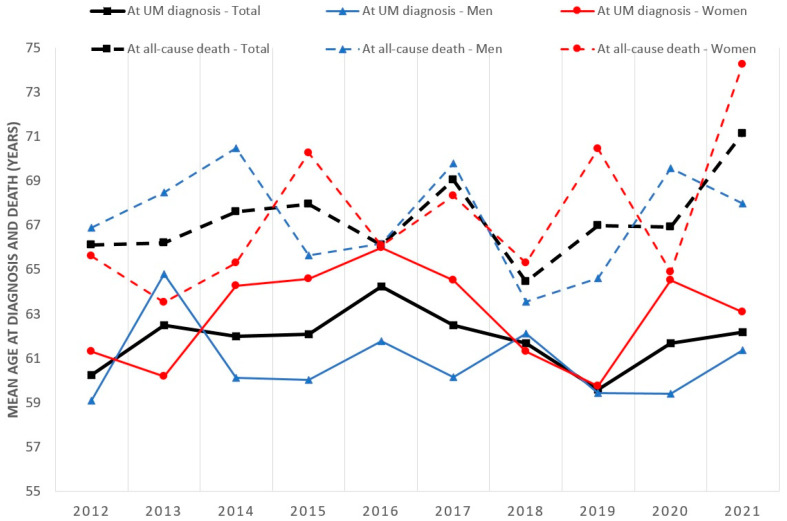
Mean age of people with uveal melanoma at diagnosis and at the time of death (all-cause mortality) in Hungary between 2012 and 2021. UM = uveal melanoma.

The crude incidence, prevalence, and all-cause mortality rates are shown in Table 1. A total of 861 patients were newly diagnosed with UM between 2012 and 2021, of whom 50.3% were women. We identified 88 and 70 new UM cases in 2012 and 2021, respectively, corresponding to 0.0011% and 0.00087% of the entire Hungarian population at risk. The proportion of newly diagnosed female patients with UM ranged between 44.6% and 58.5% between 2012 and 2021. The mean age at diagnosis of UM in male and female patients, respectively, was 59.10 ± 14.16 years (range, 20–93 years) and 61.30 ± 12.28 years (range, 33–86 years) in 2012 and 61.38 ± 15.95 years (range, 20–93 years) and 63.09 ± 11.78 years (range, 36–85 years) in 2021 (Figure 1). The total number of patients with UM fluctuated between 606 and 779 during 2012–2021, with a female predominance (53.3–57.4%).

The annual number of patients with UM who died from any cause decreased from 44 (2012) to 8 (2021). The mean age at the time of all-cause death changed from 66.11 ± 11.52 years (range, 34–93 years) to 66.94 ± 10.62 years (range, 45–87 years) from 2012 to 2021. UM was most frequently newly diagnosed in people aged 60–69 years (Table 2).

### 3.2. Incidence

**Figure 2:**
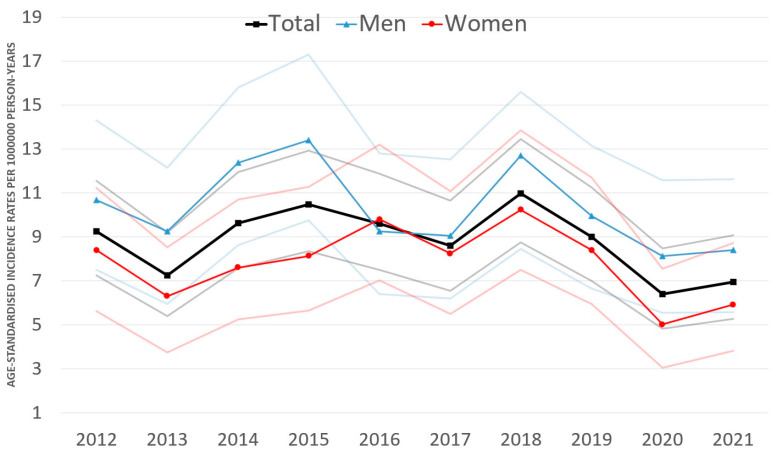
Age-standardised incidence rates (European Standard Population 2013) of uveal melanoma by gender in Hungary between 2012 and 2021 (per 1,000,000 person-years; faint lines represent 95% CI). CI = confidence interval.

The age-standardised incidence fluctuated between 6.40/1,000,000 PYs (95% CI: 4.81–8.46) and 10.96/1,000,000 PYs (95% CI: 8.75–13.45) during 2012–2021 in the total study population of Hungary (Figure 2). The annual change of age-standardised incidence of UM was not significant in either group, with rates of −2.0% (95% CI: −4.5–0.6%; *p* = 0.112) in the entire study population, −2.5% (95% CI: −5.8–1.0%; *p* = 0.160) in men, and −1.4% (95% CI: −5.0%–2.4%; *p* = 0.458) in women.

Age-standardised incidence rates were higher among men than women throughout the period analysed, except in 2016, with the lowest rate in 2020 (8.11/1,000,000 PYs; 95% CI: 5.54–11.57) and the highest in 2015 (13.38/1,000,000 PYs; 95% CI: 9.74–17.29). Cumulative age-standardised incidence was significantly higher (*p* < 0.001) in men (9.97/1,000,000 PYs; 95% CI: 8.99–11.02) compared with that in women (7.68/1,000,000 PYs; 95% CI: 6.87–8.56). The male-to-female ratio was 1.29 (95% CI: 1.12–1.51), and the proportion of men was significantly higher compared with that of women (*p* < 0.001).

### 3.3. All-Cause Mortality

Age-standardised all-cause mortality rates of UM varied between 0.79/1,000,000 PYs (95% CI: 0.27–1.63) and 5.38/1,000,000 PYs (95% CI: 3.93–7.26).

We found an 83.9% decrease (*p* < 0.001) in age-standardised all-cause mortality rates between 2012 and 2021 in the entire study population, a 78.8% decrease (*p* < 0.001) among men, and an 89.2% decrease (*p* < 0.001) in women. The annual change of age-standardised all-cause mortality was not significant in the entire study population (−15.1% annually; 95% CI: −33.6–8.6%; *p* = 0.193) or in women (−16.8% annually; 95% CI: −35.6–7.5%; *p* = 0.160), but it decreased significantly among men (−11.5% annually; 95% CI: −16.5–6.2%; *p* < 0.001).

**Figure 3:**
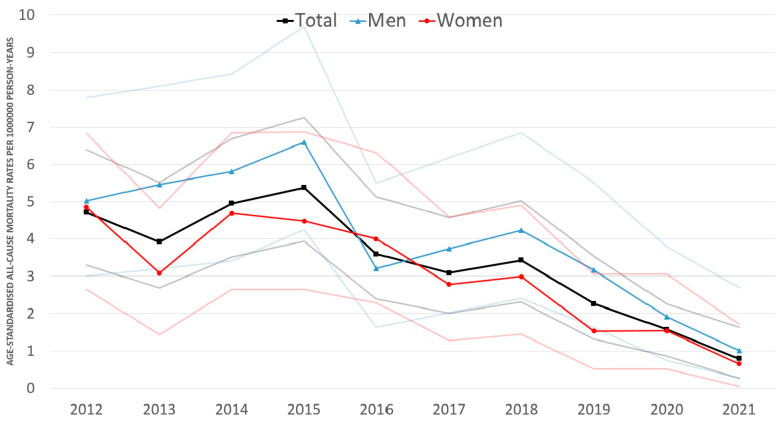
Age-standardised all-cause mortality rates (European Standard Population 2013) of uveal melanoma by gender in Hungary between 2012 and 2021 (per 1,000,000 person-years; faint lines represent 95% CI). CI = confidence interval.

Age-standardised all-cause mortality rates were higher among men than in women throughout the period analysed (except 2016) (Figure 3), with the lowest rate in 2021 (1.01/1,000,000 PYs; 95% CI: 0.28–2.70) and the highest in 2015 (6.60/1,000,000 PYs; 95% CI: 4.24–9.68). Cumulative age-standardised all-cause mortality was significantly higher (*p* = 0.008) in men (3.94/1,000,000 PYs; 95% CI: 3.34–4.63) than in women (2.86/1,000,000 PYs; 95% CI: 2.38–3.42).

## 4. Discussion

To our knowledge, this is the first study of the incidence and mortality of UM in Central and Eastern Europe. As the most frequent primary malignant tumour of the eye, understanding the epidemiological aspects of UM is important. The NHIF database is the most authoritative source of healthcare data in Hungary. Our study provides detailed information on the incidence and mortality of UM in the Hungarian population over the last decade. 

Incidence rates of UM did not change during our 10-year study; rates ranged between 6.40 and 10.96 cases per million between 2012 and 2021 in Hungary. 

The incidence rates and trends of UM vary among countries owing to different risk factors and population structures [12]. Our incidence rates are consistent with those of previous reports on the incidence of UM: 9.6 cases/million between 1960 and 2009 in Sweden [24], 6.4 cases/million between 2011 and 2017 in Canada [12], 6.4 cases/million between 2009 and 2015 in Germany [13], 6.7 cases/million between 1988 and 2007 in Israel [10], 7.0 cases/million between 1994 and 2008 in Scotland [25], 7.4 cases/million between 1943 and 1952 in Denmark [8], 6.1–7.6 cases/million between 1982 and 2014 in Australia [26,27], 8.6 cases/million in 2016 in Northern Ireland [28], 9 cases/million between 1953 and 1960 in Norway [29], 9.5 cases/million between 2010 and 2015 in Ireland [30], 10.0 cases/million between 1999 and 2010 in England [15]. However, the Hungarian incidence rates were higher than those in South Korea between 1999 and 2011 (0.4 cases/million) [31], China between 1990 and 2005 (0.6 cases/million), Japan between 2011 and 2013 (0.64 cases/million) [7], Brazil between 2010 and 2015 (4.6 cases/million) [16], the USA between 1973 and 2008 (5.1 cases/million) [11], and the USA between 2010 and 2015 (4.6 cases/million) [32]. Overall, the incidence of UM appears to be higher in Australia and Europe than in the USA or Asia. 

The incidence of UM differs between geographical regions because of differences in the ethnicities of locally settled populations. White people are more vulnerable to UM [32]. Fair skin colour and light blue eyes are risk factors for the development of UM [1]. The proportion of White people is over 90% in Hungary [33], which may explain the higher incidence rates of UM in Hungary compared with those in countries with a higher proportion of people of Hispanic, Black, or Asian races. Similar to our results, the incidence of UM was stable in Sweden [24], Australia [27], and the USA during the last 3–4 decades [11]. Interestingly, the incidence of cutaneous melanoma has also been stable in Hungary over the last decade [19], which may confute the theory that light exposure does not play a role in the development of UM [34]. In contrast to cutaneous melanoma, the role of light exposure is contentious [2]. Some reports suggested that sunlight exposure may be a risk factor for UM [35,36,37]. Long-term sun exposure appeared to increase the risk of UM in Canada and Australia [38,39]. However, the latest epidemiologic studies failed to justify an association between sunlight exposure and UM incidence [5,40].

Similar to those of previous studies [15,26,27,30,32,38], the age-standardised incidence rates of UM in our study were higher in men than in women, which may be partly associated with occupational exposure (chemical carcinogens) and diverse environmental factors (sunlight and ultraviolet and blue light) [1,2].

The mean age at the time of diagnosis varied between 59.61 years and 64.26 years during the analysed period, and these results were similar to those reported in the USA (60 years) [32], Ireland (61 years) [30], Canada (61.5 years) [38], Australia (62.8 years) [26], England (64 years) [15], and Sweden (66.0 years) [24], but higher than in China (62.8 years) [41]. The peak incidence of UM was at 60–69 years in Hungary, which is slightly lower than the ages reported in England, the USA (70–79 years) [15,32], and Sweden (75–84 years) [14]. Similar to our findings, previous studies have reported that the incidence of UM increases with age. Elderly individuals have a higher risk of developing UM [15,32]. The number of newly diagnosed cases is lower among people aged ≥80 years because the number of people aged ≥80 years is also lower in the Hungarian population.

All-cause mortality rates of UM showed a decrease during the 10-year study period, decreasing from 4.72 to 0.79 cases per million between 2012 and 2021 in Hungary. However, the decrease was most marked in the last two years, which may be attributable to the slackening of the coding discipline in Hungary because all NHIF-funded healthcare providers received an average instead of fee-for-service funding during the COVID-19 pandemic. Furthermore, in spring 2020, the Hungarian government introduced strict restrictions and lockdowns in daily life to reduce the transmission of the COVID-19 virus. Elective ophthalmic surgeries were halted and postponed, and outpatient services were only available for urgent cases for most of the year. Consequently, the lockdowns and reduced access to healthcare may have contributed to the lowest incidence rate of UM in our study period in 2020.

Similar to the rates reported by Alfaar et al. [13] in Germany, we found lower mortality rates among women than among men, which may be associated with the specific hormonal profiles and varied genetic predispositions of women [42,43,44]. 

Despite advancements in ultrasound imaging, local eye-sparing treatments, and systemic medications for metastatic UM, disease-specific survival has not improved over the past 40 years [30,45]. No evidence for the effectiveness of therapeutic options for metastatic UM is available [46]. About half of patients with UM develop metastases [47]. The overall survival of patients with metastatic UM is estimated to be 1.07 years [46]. The development of local therapies has not improved metastatic rates [11]. New treatment modalities for metastatic cutaneous melanoma are not beneficial for metastatic UM [46]. Metastatic UM is remarkably resistant to immunotherapy, potentially due to processes related to 2,3 dioxygenase [48]. Furthermore, nitric oxide synthase expression is associated with poor prognosis and reduced survival in UM [49]. Despite improvements in treatment options, five-year disease-specific survival remains unchanged, as reported in the USA (80.9%) between 1973 and 2013 [50], Australia (81%) between 1982 and 2015 [27], Northern Ireland (82.6%) in 2016 [28], and China (86.6%) [41].

In Hungary, brachytherapy using ruthenium-106 plaques is the only available eye-sparing procedure for UM. Transpupillary thermotherapy, owing to its controversial nature, is available as a supplemental treatment along with ruthenium-106 brachytherapy in patients with choroidal melanoma [51]. Recurrence after transpupillary thermotherapy in patients with UM is approximately 9–28% [52]. The fundamental requirements for the effective management of UM encompass timely detection, the formulation of a therapeutic plan, and the provision of access to suitable treatments [53].

During the analysed period, there were no changes in the management or treatment of UM in Hungary. The decrease in all-cause mortality may be related to the improvement in general health status, health literacy, and general medical care and increased participation in screening programmes compared with UM-specific medical care. For instance, cardiovascular diseases are among the most common causes of death in Hungary, and cardiovascular disease-related cause-specific mortality decreased from 803/100,000 to 543/100,000 from 2000 to 2019 [54]. 

This study has some limitations. Surgically treated iris melanoma cases, untreated UMs, and UM cases treated outside Hungary (iodine-125 brachytherapy and proton beam therapy are not available in Hungary) were not included in our sample, which may have caused a slight underestimation of the incidence of UM. The inclusion of some cases of choroidal metastasis and raised choroidal nevi treated with brachytherapy in our sample could have resulted in a minor overestimation of the incidence of UM. Moreover, the NHIF database contains no information about patients’ ethnicity; frequency distribution regarding the iris, ciliary body, and choroidal melanoma; clinical stage; or cause-specific mortality.

## 5. Conclusions

The incidence of UM in Hungary is comparable to that in other European countries, and the incidence rate did not change markedly during the analysed period. All-cause mortality rates showed a decreasing trend, but this decline could not be attributed to improved treatment modalities for the primary tumour and metastatic UM. Higher incidence and mortality rates were found in men than in women. Our study contributes to our understanding of the epidemiology of UM in Central and Eastern Europe. Our findings close a gap in ocular oncology in Hungary, facilitate comparisons between different countries and regions, and provide useful baseline data for analysing future changes.

## Figures and Tables

**Table 1:** Crude numbers, incidence, prevalence, and all-cause mortality of uveal melanoma in Hungary between 2012 and 2021.

	2012		2013		2014		2015		2016		2017		2018		2019		2020		2021		Total	
	N	%	N	%	N	%	N	%	N	%	N	%	N	%	N	%	N	%	N	%	N	%
**Incidence**																						
Total (N, % of population at risk)	88	0.0011	70	0.00086	93	0.0011	100	0.0012	94	0.0012	84	0.0010	108	0.0013	89	0.0011	65	0.00081	70	0.00087	861	0.0010
Men	42	47.7	35	50.0	51	53.8	55	55.0	39	41.5	39	46.4	51	47.2	43	48.3	36	55.4	37	52.9	428	49.7
Women	46	52.3	35	50.0	42	46.2	45	45.0	55	58.5	45	53.6	57	52.8	46	51.7	29	44.6	33	47.1	433	50.3
**Prevalence**																						
Total (N, % of total population)	669	0.0082	606	0.0074	666	0.0082	697	0.0086	726	0.0089	731	0.0090	771	0.0096	779	0.0097	634	0.0079	655	0.0082	6934	0.0084
Men	288	43.0	258	42.6	312	46.8	309	44.3	331	45.6	335	45.8	361	46.8	364	46.7	302	47.6	304	46.4	3164	45.6
Women	381	57.0	348	57.4	354	53.2	388	55.7	395	54.4	396	54.2	410	53.2	415	53.3	332	52.4	351	53.6	3770	54.4
**All-cause mortality**																						
Total (N, % of prevalent population)	44	6.58	37	6.11	47	7.06	50	7.17	35	4.82	29	3.97	33	4.28	24	3.08	16	2.52	8	1.22	323	4.66
Men	17	38.6	20	54.1	21	44.7	25	50.0	12	34.3	14	48.3	16	48.5	13	54.2	7	43.8	4	50.00	149	46.1
Women	27	61.4	17	45.9	26	55.3	25	50.00	23	65.7	15	51.7	17	51.5	9	45.8	9	56.2	4	50.00	174	53.9

**Table 2:** Aggregated age and gender composition of people with new uveal melanoma in Hungary between 2012 and 2021.

Age Group (Years)	Men		Women		Total	
	N	%	N	%	N	%
18–29	9	2.1	5	1.2	14	1.6
30–39	30	7.0	29	6.7	59	6.9
40–49	56	13.1	48	11.1	104	12.1
50–59	82	19.2	73	16.9	155	18.0
60–69	123	28.7	124	28.6	247	28.7
70–79	92	21.5	110	25.4	202	23.5
80–89	33	7.7	43	9.9	76	8.8
90+	3	0.7	1	0.2	4	4.6
Total	428	100	433	100	861	100

## Data Availability

Data will be made available upon request from the corresponding author.
